# Biological Role, Mechanism of Action and the Importance of Interleukins in Kidney Diseases

**DOI:** 10.3390/ijms23020647

**Published:** 2022-01-07

**Authors:** Paulina Mertowska, Sebastian Mertowski, Iwona Smarz-Widelska, Ewelina Grywalska

**Affiliations:** 1 Department of Experimental Immunology, Medical University of Lublin, 4A Chodzki Street, 20-093 Lublin, Poland; paulinamertowska@umlub.pl (P.M.); ewelina.grywalska@umlub.pl (E.G.); 2 Department of Nephrology, Cardinal Stefan Wyszynski Provincial Hospital in Lublin, Al. Kraśnicka Street, 20-718 Lublin, Poland; i.widelska@interia.pl

**Keywords:** chronic kidney disease, acute kidney disease, cytokine, kidney transplantation

## Abstract

Each year, the number of patients who are diagnosed with kidney disease too late is increasing, which leads to permanent renal failure. This growing problem affects people of every age, sex and origin, and its full etiopathogenesis is not fully understood, although the involvement of genetic susceptibility, infections, immune disorders or high blood pressure is suggested. Difficulties in making a correct and quick diagnosis are caused by the lack of research on early molecular markers, as well as educational and preventive activities among the public, which leads to the late detection of kidney diseases. An important role in the homeostasis and disease progression, including kidney diseases, is attributed to interleukins, which perform several biological functions and interact with other cells and tissues of the body. The aim of this article was to systematize the knowledge about the biological functions performed by interleukins in humans and their involvement in kidney diseases development. In our work, we took into account the role of interleukins in acute and chronic kidney disease and kidney transplantation.

## 1. Introduction

Interleukins are a group of protein compounds belonging to a type of cytokines that are produced by numerous cells in the body, including immune cells. They are involved in several important cellular processes, including proliferation, maturation, migration and adhesion, and also participate in the activation and differentiation of immune system cells [1,2,3]. Currently, according to the literature, we have distinguished about 40 interleukins, and their number is constantly growing (at the end of the 20th century, this group consisted of 30) [4]. Moreover, it has been shown that interleukins as an element of the cytokine network affect the entire human organism, including metabolic activity and cardiovascular and neuroendocrine systems, allowing the maintenance of homeostasis [5]. However, their complex and broad spectrum of actions and influence on other cells may lead to their over-depression and the development of disease. This regards autoimmune diseases, diabetes and neoplastic and neurological disorders, as well as kidney diseases [4,6,7]. However, despite the importance of interleukins, their routine evaluation is not a widespread gold diagnostic standard in modern medicine. From a clinical point of view, their level is determined only in severe conditions, including septic shock, disseminated intravascular coagulation or respiratory failure (according to the literature data, IL-6, TNF-*α* and IL-1 *β* are responsible) [8,9].

Kidney disease is one of the growing challenges for modern medicine, as there are many potential causes of the development of the aforementioned diseases, which include genetic susceptibility, the development of infections, immune disorders, diabetes and high blood pressure. Due to this variety of potential causes, the diagnosis of kidney disease is difficult [10]. Currently, kidney disease is classified into two types: acute kidney injury (AKI), defined as a transient loss of kidney function lasting less than three months in response to injury, infection or medication, and chronic nephritis, which is a consequence of untreated kidney disease [11,12]. The statistics show that kidney disease affects many people around the world, and its progression depends on gender, age and even race or place of residence. A 2017 report showed that the incidence of chronic kidney disease (CKD) was nearly 8724 cases per 100,000 inhabitants. Detailed data presented by the researchers referring to individual regions of the world in the context of CKD incidence showed that the highest was observed among people living in Eastern Europe (12,408 cases per 100,000), while the lowest among Western European residents (5446 cases per 100,000) [13]. Such a discrepancy may result from malfunctions of healthcare systems in selected regions of the world, as the research conducted by the CDC in 2021 on US residents showed that nearly 90% of people with CKD are not aware of the disease [14]. This was also confirmed by the results of a Polish report published in 2019, which showed that only 15% of nephrological patients are in the first stage of the disease, and the largest group of patients are in stage IV/V of kidney disease [15]. Globally, renal diseases are detected very late and most often when patients are hospitalized for the treatment of another disease. This has led to the inclusion of kidney failure to the list of civilization diseases as a consequence of the lack of early diagnosis (based on molecular markers), as well as educational and preventive actions in society [12].

The assessment of the levels of interleukins in the human body can be used as a diagnostic indicator of the development or progression of many diseases (cancers [16,17,18,19], heart disease [20,21,22] and neurological disorders [23,24,25]) affecting contemporary society, including kidney diseases [26,27,28,29,30]. That is why it is extremely important to conduct further extensive scientific and clinical research to assess the biological functions of interleukins and their interactions with other cells of the body, which may lead to health deterioration. The aim of this article was to systematize the knowledge about the biological functions performed by interleukins and their involvement in the development of renal disfunctions. In our work, we considered the role of interleukins in acute and chronic kidney disease and kidney transplantation.

## 2. Characteristics, Biological Role and Mechanism of Action of Interleukins in the Human Body

**Figure 1:**
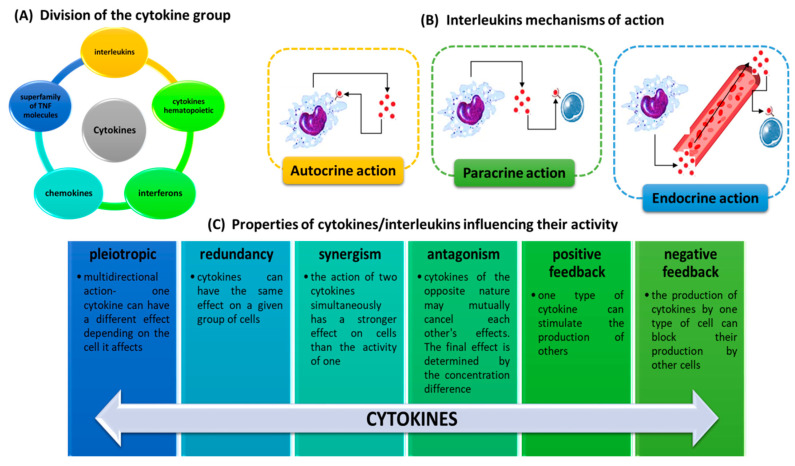
Characterization of interleukins. (**A**) Division of the cytokine group. (**B**) Mechanisms of action of interleukins. (**C**) Properties of interleukins influencing their activity (based on [5,31,32,33,34]).

Interleukins, apart from interferons and chemokines, belong to a group of cytokines (Figure 1A) showing very complex and broad actions in the human body, interacting with all cells of the immune system and other cells and tissues and creating a network of connections—a cytokine network [31]. These glycoprotein signal molecules have several properties that influence their activity. These include pleiotropicity, redundancy, synergism, antagonism and positive and negative feedbacks (Figure 1C) [32,33,34]. Like cytokines, interleukins exhibit three characteristic mechanisms of action on other cells: autocrine (the substance affects the cell that produces it), paracrine (the substance affects tissues close to the cell that produces it) and endocrine (the substance produced by the cell enters the bloodstream and is transported to distant organs) (Figure 1B) [5].

### 2.1. Classification of Interleukins

The classification of interleukins is extremely diverse, as is the very group of these immunomodulating protein molecules. In the literature, we can find the division of interleukins based on the way they interact with lymphocytes, the structure of the molecules themselves, the structures of the receptors for these molecules, their functions and proinflammatory or anti-inflammatory properties [2,35]. Currently, one of the most frequently used methods of dividing interleukins is their classification for class I and II cytokines, which depends on the structure of these molecules. Class I cytokines are characterized by having a core composed of four tightly packed *α*-helices arranged in an “up–down–down” orientation. In contrast, class II cytokines are characterized by a similar structural motif using six to seven *α*-helices. In addition, there are some specific features and motifs in the structures of interleukins that allow them to be grouped even within class I or II [2]. Class I cytokines can be divided into two groups based on the lengths of the cores forming the bundle into long chains (where the lengths of the internal bundles affect the lengths of the entire peptides, this applies to interleukins above 165 amino acids, e.g., IL-6, IL-11, IL-12A, IL-23A, IL-27A and IL-31) and short chains (up to 165 amino acids in length per molecule, e.g., IL-2, IL-3, IL-4, IL-5, IL-7, IL-9, IL-13, IL-15 and IL-21) [2,36,37,38,39].

Another way to classify interleukins is their division based on their similarity in terms of the structure and location of genes encoding individual proteins and considering their first- and second-order structures together with the complexes of the used receptors. This broad classification allowed for the selection of five families of interleukins. The IL-1 family was created according to gene similarity, and in addition, it performs similar functions to the Toll-like receptor (TLR) family and is associated with innate immunity. We include, in this case, such interleukins as: IL-1, IL-18, IL-33, IL-36, IL-7 and IL-38 [40]. Another family is the IL-6 family, the division of which is based on a common signaling receptor subunit, the 130-kDa glycoprotein (gp130). We include here such interleukins as: IL-6, IL-11 and IL-31 [41]. The interleukins belonging to the IL-10 family are mainly monomeric and contain one or more α-helices in their upper loops, including IL-10, IL-19, IL-20, IL-22, IL-24, IL-26 and IL-28 [42]. The next family is the IL-12 family, which includes heterodimeric glycoproteins composed of the *α* and *β* chains subunit. This results in unique characteristics of the group that allow them to create a set of connections and interactions that other families or interleukins do not have. This group contains such molecules as: IL-12, IL-23, IL-27, IL-30, IL-35 and IL-39 [43]. Another family is the IL-17 family, that has been distinguished according to the homology of the amino acid sequences, including IL-17, IL-17A (CTLA-8), IL-17B, IL-17C, IL-17D, IL-17E (IL-25) and IL-17E [44].

**Figure 2:**
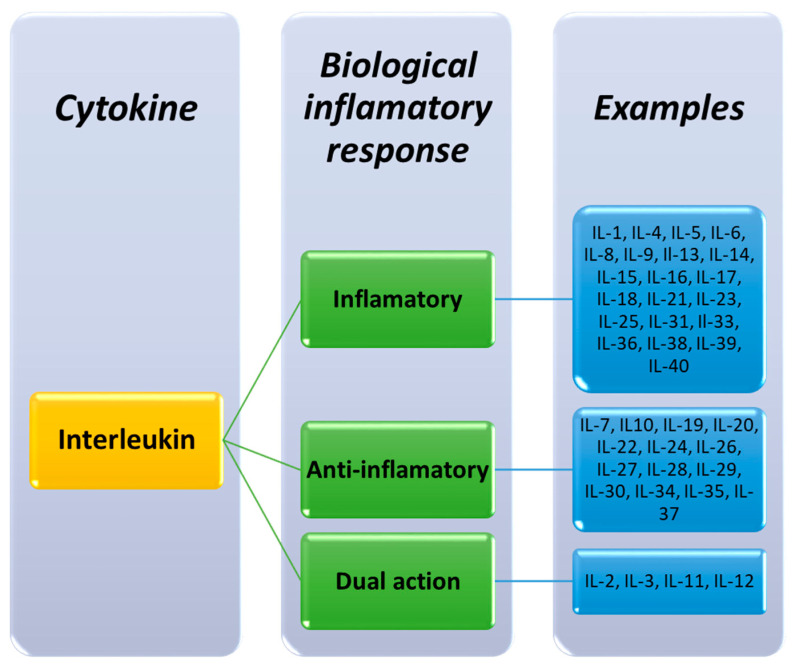
Classification of interleukins based on their biological inflammatory response effects [4].

Another method for the classification of interleukins adopted in the literature was the division based on their biological effect in the inflammatory response. There are three groups distinguished. The first, largest group are inflammatory cytokines, which include 22 molecules, such as IL-1, IL-4, IL-5, IL-6, IL-8, IL-9, IL-13, IL-14 and IL-15. The second group includes anti-inflammatory molecules, which are 14 interleukins, such as: IL-7, IL-10, IL-30 and IL-37. The last group consists of interleukins with a dual function, which, in appropriate situations, can act as inflammatory and anti-inflammatory molecules—IL-2, IL-3, IL-11 or IL-12 (Figure 2) [4].

### 2.2. Molecular Characterization of Interleukins

Molecularly, interleukins are an extremely diverse group of proteins ranging in length from 99 (IL-8) to 1332 amino acids (Pro-IL-16) (Table 1). This also applies to their molecular weight (11.098–141.752 kDa), isoelectric point (from 4.41 for IL-18 to 10.5 for IL-11) and the contents of the hydrophilic and hydrophobic amino acids in the composition of a molecule (Table 1). Most of the interleukins are monomeric compounds, with the exception of IL-35, which is composed of the subunits IL-12*α* and IL-27*β*, and IL-39, composed of IL-23p19 and Ebi3 (Epstein–Barr virus-induced gene) subunits [45]. Detailed characterization of the interleukins based on the aforementioned properties, as well as the analysis of the secondary structure, is presented in Table 1. Despite the structural and functional similarities based on the use of the same receptors, individual groups or families of interleukins, their amino acid sequence similarities are rather low. The bioinformatic analyses of the amino acid sequences of the selected interleukins carried out by our team showed that, depending on the family, it varied: 6–23% identity for the IL-1 family (except for IL-37, whose amino acid sequence identity between IL-1 is as high as 97%), 7–20% for the IL-6 family, 5–27% for the IL-10 family and 7–19% for the IL-12 family (Supplementary Materials Table S1). When analyzing the amino acid sequence identity of inflammatory interleukins, the range of amino acid sequence identity was 4–26%. Among the interleukins belonging to the group of anti-inflammatory cytokines, the identity of the amino acid sequences ranged from 4 to 36%. For individual interleukins within this group, the amino acid sequence identity range was as follows: for IL-7: 6–15%, for IL-10: 8–23%, for IL-19: 7–36%, for IL-20: 5–36%, for IL-22: 6–21%, for IL-24: 8–27%, for IL-26: 4–23%, for IL-27α/IL-30: 7–17%, for IL-28: 5–10%, for IL-29: 7–17%, for IL-34: 4–15% and for IL-37: 7–13%. Within the group of double-acting interleukins, the amino acid sequence identity was, for IL-2: 13–16%, for IL-3: 11–16%, for IL-11: 13–16% and for IL-12: 11–16%. This means that, within this group of interleukins, the range of amino acid sequence identities ranged from 11% to 16%.

Interleukins, like the whole group of cytokines, differ in their activity and functions. It is a consequence of the diverse process of gene regulation during the synthesis of individual molecules and, as indicated in the literature, by the contribution of environmental factors. The UNIPORT system databases contain information on the influence of point mutations on the activity and properties of interleukins. So far, changes in the activity profile have been demonstrated, resulting from changes in the amino acid sequences for four interleukins: IL-6, IL-17A, IL-18 and IL-33 [51,63,64,80]. The most/mutations, as many as 20 mutation sites, were recorded for IL-18, while, for the remaining three molecules, five. Most of the mutations that occur have their consequences in the form of a decrease in activity or affinity for a specific receptor corresponding to the interleukin understudy, as well as changes in the functions performed. A detailed analysis of the mutation sites and their effects is presented in Table 2.

### 2.3. Origin and Biological Functions of Interleukins in Health and Disease

Despite having many structural features and molecular partners in common, interleukins mediate surprisingly diverse functional effects. It is possible thanks to the influence on many cells of the immune system, including lymphocytes, macrophages, NK cells, neutrophils, eosinophils, basophils, hematopoietic cells, endothelial cells and keratinocytes. This enables them to be involved in many cellular processes, such as proliferation, maturation, activation, chemotaxis and phagocytosis. However, as we mentioned in the introduction, apart from the beneficial effects of interleukins for our body, they can also cause the emergence and progression of many disease states. This mainly applies to the state in which the production of specific interleukins is too high. Interleukin overexpression may lead to the development of asthma, allergies and autoimmune diseases, as well as neoplastic diseases such as: chronic lymphocytic leukemia or multiple myeloma. A detailed analysis of the origin, target cells for interleukins and their effects under physiological conditions, as well as their arrangement in particular disease states, is presented in Table 3.

## 3. The Importance of Interleukins in Kidney Diseases

**Figure 3:**
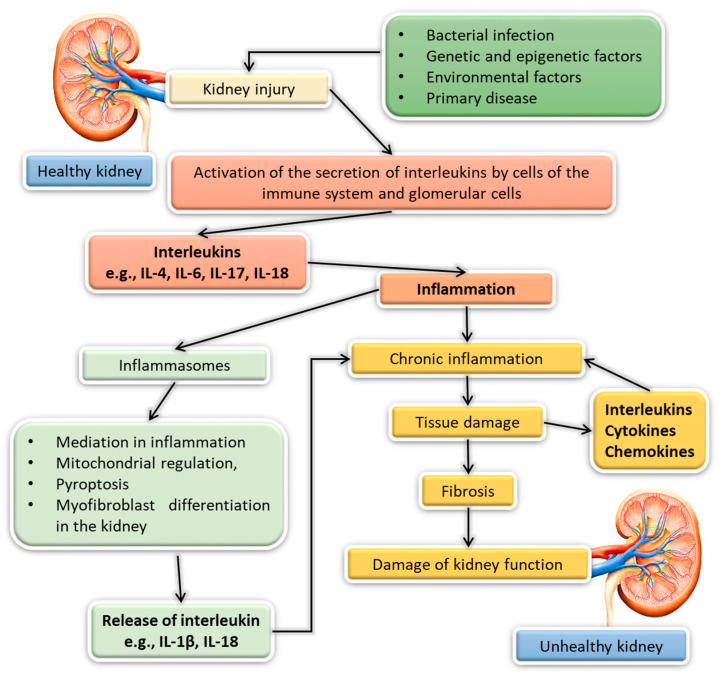
Influence of interleukins on the development of chronic inflammation and, consequently, kidney damage [173,178,188].

In previous sections, we wrote about the importance of interleukins in maintaining the homeostasis of the immune system and the body’s defense in pathological conditions. Interleukins play an extremely important role in modulating the immune response, because they influence various effector pathways that may affect the development of inflammation [173]. It is one of the mechanisms in which our body and, more specifically, the immune system deals with a pathological situation caused by various external and internal stimuli, including the presence of viruses, bacteria, foreign bodies or the development of necrosis, as well as unfavorable chemical factors. The main purpose of inflammation is to reduce the harmful factor and then neutralize it and repair damaged tissues [174]. Sometimes, the immune reaction of our body is inadequately intensified in relation to the damaging stimulus or is directed against healthy tissues. Then, there is also the development of inflammation in the human body, which, in turn, may lead to other diseases, such as: allergic and autoimmune disorders, as well as kidney diseases (Figure 3) [175,176]. In the human body, including the kidneys, inflammation can take two forms, depending on its duration. The first form is acute inflammation that lasts up to several days and consists of three main stages: tissue damage, increased blood flow and influx of immune cells (exudate), and healing. The second one is chronic inflammation that lasts for many weeks. In its course, all three stages of inflammation take place simultaneously, and the chronic activity of immune cells leads to gradual damage of the affected tissues and their fibrosis (Figure 3). Inflammation is closely related to kidney disease and involves a complex network of interactions between kidney parenchymal cells and the immune cells found in the kidneys (such as macrophages and dendritic cells and circulating monocytes, lymphocytes and neutrophils) [176,177]. When stimulated, these cells activate specialized structures such as pattern recognition receptors (PRRs) that trigger major pathways of innate immunity (including inflammation), causing metabolic reprogramming and phenotypic changes in immune and parenchymal cells and triggering the secretion of several inflammation mediators (cytokines, chemokines and acute phase proteins) that can cause irreversible tissue damage and loss of function [178]. PRRs include pathogen-related molecular pattern recognition receptors (PAMPs) and danger-related molecular patterns (DAMPs) that are common to pathogens [179]. Kidney-specific DAMPs include Tamm-Horsfall glycoprotein or uromodulin, which are released by tubular damage, while non-specific DAMPs consist of intracellular particles, including histones, a group 1 highly mobile protein (HMGB1), and parts of the cytosol. DAMPs induce innate immunity by activating the NRLP3 inflammasome, G protein-coupled class receptors or the Toll-like receptor (TLR) [179,180]. The mechanism of the inflammation development is an extremely complicated, multistage process that requires the coordination of many factors and proteins, including interleukins. As indicated in the literature, currently, only a few different pathways of the inflammatory response are known, such as: p38 mitogen-activated protein kinase (p38 MAPK), interleukin-6 (IL-6)/Janus kinase (JAK)/signal transducer and transcription activator 3 (STAT3) and phosphoinositide 3-kinase (PI3K) [178]. Another way that interleukins are involved in the development of inflammation in kidney disease is through the formation of inflammasomes. These are macromolecular complexes known from the proteolytic pathway of caspase 1 activation, which, in addition to mediating the development of inflammation, are also involved in the process of proptosis (lytic cell death), mitochondrial regulation and myofibroblast differentiation [181]. Examples of inflammasomes are NOD (nucleotide oligomerization domain), LRR (leucine-rich repeats) and protein 3 containing a pyrin domain (NLRP3) [182,183,184]. Their activation in phagocytes and kidney podocytes causes the release of IL-1 and IL-18, which may cause inflammation [185,186]. Additionally, NLRP3 has been implicated in renal fibrosis, diabetic nephropathy, obesity-related kidney disease, chronic glomerulonephritis, immunoglobulin A (IgAN) nephropathy, crystalline nephropathy and hyperhomocysteinaemia-induced kidney damage [178]. In addition, interleukins can be secreted both by lymphocytes and internal kidney cells (glomerular, endothelial, tubular or mesangial cells), and the very increase in their level is associated with the progression of nephropathy, which may be an important factor affecting the course and progression of kidney disease [173,187]. (Figure 3). In connection with the above-mentioned information, in this review, we would like to present their importance in the pathogenesis of selected kidney diseases.

### 3.1. Acute Kidney Injury

AKI, also called acute renal failure, leads to a sudden loss of normal kidney function. This rapid reduction in the function of these organs is associated with an increase in the blood creatinine levels accompanied by a decrease of urine output below 500 mL/day. This condition adversely affects both the short-term and long-term survival of the patient due to the greater risk of developing chronic and end-stage renal disease, as well as a cardiovascular event. Research data suggest that the occurrence of AKI is one of the risk factors for increased mortality not only in adult patients but also in children [189]. The epidemiology of AKI depends on the selected population and the adopted criteria of diagnosis and laboratory tests specific to a given country. In addition to changes in the levels of urea nitrogen and creatinine in the bloodstream and in the amount of urine excreted, the water–electrolyte and acid–base balances deteriorate in patients, which may contribute to the need for the hospitalization of such people [190,191]. In the case of AKI in children, clinical trials have shown that its occurrence may be associated with a higher risk of proteinuria and arterial hypertension [29,190]. Therefore, several scientists are conducting research aimed at finding new biomarker molecules that would enable the early diagnosis of AKI [28]. Currently, the levels of creatinine and blood urea nitrogen (BUN) are used as marker molecules, but they are not sensitive enough to allow early diagnosis of the disease, as their levels are only visible in cases of significant kidney damage. In addition, the level of these markers in the blood may also be influenced by other factors, such as: malnutrition, infections, concomitant medications and even the patient’s gender [28]. Therefore, we are looking for other molecules that can be used in the future as potential markers of disease development. One example of such molecules could be interleukins.

In the case of AKI, one of the molecules involved in its development may be IL-18, which is responsible for the induction of interferon-gamma. It is also involved in the regulation of innate and acquired immunity. IL-18 can be produced in the form of an inactive precursor by many cells, such as: proximal tubular epithelial cells and intercalated collecting cells, monocytes and macrophages [192]. It is activated by caspase 1. Activated IL-18 exerts a proinflammatory effect through signal transduction by the helper protein heterodimer of the IL-18 receptor. The IL-18 levels are increased in many endogenous inflammatory processes, such as sepsis, and numerous studies have indicated IL-18 as both a mediator and a biomarker of AKI. Its level increases approximately 6 h after ischemic injury, 24–48 h before AKI diagnosis, and peaks approximately 12 h later to values up to 25 times more than normal [192]. However, despite the above information, the picture of IL-18 as a predictor may not be clear. In the studies conducted by Nisula et al., the conclusions drawn from the analyses indicate that IL-18 may have a weak or moderate predictive value for AKI, RRT (renal replacement therapy) or 90-day mortality in ICU patients. Therefore, this role as a biomarker of the early diagnosis of AKI should still be investigated and cannot be used for the unambiguous prediction of AKI [193].

Another interleukin that deserves attention is IL-20, which may be a therapeutic agent for AKI. The mechanism of its interaction is based on three receptors: IL-20R1, IL-20R2 and IL-22R1. Studies using animal models have shown that the level of IL-20 and its receptors increased in response to IRI and HgCl2, confirming the involvement of this interleukin in the progression of AKI. Additionally, IL-20 has been shown to increase the expression of TGF-*β*1 and to promote cell death (by activating caspase 9 in proximal tubular epithelial cells (HK-2)). Therefore, researchers have suggested that this cytokine may be associated not only with tubular cell death but also with tubule interstitial fibrosis and nephritis seen in AKI progression. Laboratory studies have shown that IL-20 expression demonstrated a similar upward trend as the serum creatinine and BUN levels, which may indicate the participation of IL-20 in the progression of AKI. All these changes in the kidney related to renal cell apoptosis, fibrosis and the development of inflammation are important factors in the transition from AKI to CKD [194,195].

In the literature, we can also find information about the role of IL-6 in the pathogenesis of AKI. This cytokine can be produced by cells residing in the kidneys (including tubular epithelial cells, podocytes and mesangial cells) under the influence of stimuli such as TNF-*α* and IL-1*β*. Moreover, IL-6 is involved in several cellular processes within the kidneys, such as induced collagen I expression in mouse proximal tubular epithelial cells, participation in mesangial cell proliferation (glomerular hypertrophy and stimulation of MCP-1 expression) and podocyte apoptosis (as a result of induction with high glucose levels). It also increases the production of p21 and p27 (causes cycle arrest) cell in podocytes. Studies have shown that IL-6 deficiency in AKI patients improves kidney function and reduces neutrophil infiltration. This has also been confirmed in the results of animal models, where the blockade of IL-6 resulted not only in the improvement of kidney function by reducing the production of TNF-*α* and IL-1*β* but also contributed to the reduction of ICAM-1 and P-selectin expression, which are associated with the process of infiltration of neutrophils. Based on these data, it has been suggested that IL-6 not only contributes to the development of inflammation in the kidneys, which results in the deterioration of the functional processes of these organs but also participates in the disruption of the structure of the glomeruli [196,197,198].

However, recent studies have indicated that the role of IL-6 in the development of kidney disease is not fully understood. It has been shown that the participation of this cytokine in kidney diseases may also play an anti-inflammatory role. This is indicated by studies in which the enhancement of the IL-6/sIL-6 axis protects against the development of AKI induced by HgCl2 due to the reduction of oxidative stress in cells. According to some researchers, this indicates a protective role of IL-6 against nephrotoxic nephritis. Due to these two different views on the role of IL-6, it seems important to undertake new research aimed at a more detailed understanding of the function of this cytokine in renal dysfunction [194,199,200,201].

Another interleukin that plays an important role in the pathogenesis of AKI is IL-10. In the human body, IL-10 acts as an anti-inflammatory cytokine, responsible for limiting the development of inflammation and inhibiting inflammatory reactions by reducing the secretion of proinflammatory cytokines and inhibiting the immune system cells (including lymphocytes, dendritic cells, NK cells and macrophages) [29,202]. Genetic studies have shown that the presence of several single nucleotide polymorphisms in the IL-10 promoter region is associated not only with the faster deterioration of kidney function but also with the development of glomerulonephritis (IgA nephropathy (IgAN) and focal segmental glomerulosclerosis (FSGS)) [203]. Studies on animal models have shown that disturbances in the IL-10 levels significantly increase proteinuria and reduce GFR [203,204]. The first mention of the use of IL-10 as a biomarker appeared in 2015 in studies conducted by Greenberg et al., in which they showed IL-6 and IL-10 as AKI biomarkers after pediatric cardiac surgery [29].

### 3.2. Chronic Kidney Disease

CKD affects 8–16% of the world’s population [205]. By definition it is a disease resulting from progressive damage of the kidneys manifested by abnormal albumin excretion or the deterioration of renal function, quantified on the basis of the tested glomerular filtration rate (GFR) [206] (GFR below 60 mL/min/1.73 m^2^ and albuminuria of 30 mg per 24 h or markers of kidney damage such as hematuria and structural abnormalities) persisting for more than 3 months are the basic factors confirming CKD [205,206]. In the case of CKD, its development may depend on many factors; however, the studies conducted so far have shown a significant importance of IL-6. The plasma levels of IL-6 are increased in patients with CKD [198]. IL-6 may be involved in fibrosis and tissue damage, as identified in angiotensin II-induced renal disease models. In this case, angiotensin II infusion induces the expression of IL-6 leading to renal fibrosis, while, in IL-6 deficient mice, they remain resistant to kidney damage [207]. Another IL-6 effector mechanism may be to enhance the signaling response of epithelial cells to factors such as cytokines and transforming growth factor-*β* (TGF-*β*) [207].

As indicated in the literature, another interleukin involved in development may be IL-20, the level of which was elevated in the serum of CKD patients. Additionally, IL-20 was expressed in the kidneys, heart, liver and lungs of the rat CKD model after 5/6 nephrectomy and in the interstitial immune cells and glomerular mesangial cells of CKD rats [208]. It has also been shown that IL-20 is able to induce apoptosis in mouse renal epithelial cells and is able to increase TGF-*β*1 production by rat interstitial fibroblasts. The above-mentioned cell types have also been found to be involved in the pathogenesis of CKD [209]. It is also important to note that the variability of the IL-20 levels may be related to the severity of kidney damage in CKD patients, but this thesis requires further analysis [209].

Another interleukin involved in the development of CKD is IL-1. As evidenced by the results of studies conducted in research models in IL-1 receptor antagonist, CKD knockout mice were also found to be anemic, and the researchers found that the degrees of anemia and kidney damage were modulated (both worsened and improved) by the degree of IL-1 expression. In studies, IL-1 receptor antagonist (RaKO) knockout mice showed elevated levels of circulating leukocytes, hepatic CRP, hepatic IL-1*β*, renal TNF-*α*, IL-10 and IL-6 compared to mice with adenine diet-induced CKD. Renal deterioration was also found based on symptoms such as fibrosis, tubular atrophy dilatation, tissue damage and macrophage infiltration. Interleukin IL-1 itself plays an important role in acute and chronic inflammation, host defense and acute-phase responses, enhancing the infiltration of inflammatory cells and increasing the expression of adhesion molecules [210].

The cause of the development of CKD is the presence of many other diseases leading to progressive loss of normal kidney function, including the development of glomerulonephritis (membranous nephropathy (MN), IgAN, FSGS, diabetic nephropathy (DN) or kidney damage as a result of arterial hypertension (hypertensive nephropathy).

#### 3.2.1. Membranous Nephropathy (MN)

MN is an autoimmune disorder that develops as a result of the build-up of immune complexes along the subepithelial region of the glomerular basement membrane [211]. One of the symptoms of MN is an elevated level of protein in the urine, associated with a pathognomonic pattern of glomerular damage [212]. In the case of MN, IL-4 is probably involved in its pathogenesis. In studies conducted by Masutani et al. on patients with idiopathic membranous nephropathy (IMN), it was shown that there is a relationship between the percentage of single IL-4-positive cells and serum IgG levels; in a group of MN patients, those with high IL-4 production had low IgG levels. Moreover, the group of these patients presented full-blown nephrotic syndrome [213]. However, the same team also found that the percentage of IL-4-positive cells correlated significantly with daily protein excretion, which may suggest that the overproduction of IL-4 may affect the rate of protein excretion in the urine [213]. Giacomelli et al. confirmed the involvement of IL-4 in the pathogenesis of IMN [214]. IMN has been shown to be a nephritogenic immune disorder associated with type 2 (Th2) helper T-lymphocytes, which are responsible for the increased production of IL-4. Interleukin IL-4 itself induces isotype switching towards IgG4 and IgE, and this process is enhanced by another cytokine produced by Th2 IL-10 [215,216,217]. The task of IL-10, which acts on B-lymphocytes, is to stimulate the production of antibodies. Research confirms that IL-10 is also closely related to IL-4. The stimulation of IL-10 peripheral blood mononuclear cells (PBMC) has been shown to increase IL-4-induced *γ*4 transcription and IgG4 production [217]. In addition, it has been shown by Giacomelli et al. that the presence of genetic polymorphisms within IL-10 is associated with an increased CD4/CD8 ratio in the development of IMN [214,218]. It was shown that, in these patients, the level of IL-10 in urine was higher compared to patients with a CD4/CD8 ratio of less than two. Therefore, scientists postulated that the CD4/CD8 ratio of IL-10 may be strongly related to the pathogenesis of IMN [214,219]. In the literature, we can also find a brief reference to the influence of IL-5 on the development of MN. Its role was presented in the results of the research conducted by Ifuku et al. It has been found that urinary IL-5 may be related to glomerular immunological phenomena. This conclusion was confirmed by the higher concentrations of IL-5 and IL-4 observed in patients with MN compared to the other research groups analyzed in this study [220]. IL-17 may be another cytokine involved in the pathogenesis of MN. Rosenzwajg et al. showed a reduction in the IL-17A levels in patients diagnosed with MN, and it has been suggested that such a condition may be related to the leakage of proteins in the urine in these patients [221]. Another study by Li et al. suggested an increased Th17 immune response in MN patients, which was detected in an intracellular cytokine production assay, when stimulated with a leukocyte-activating cocktail [222]. The studies conducted by Cremoni et al. also showed a significant role of IL-17 in the pathogenesis of MN. It has been found that, among 53% of patients diagnosed with MN, disease progression is mediated by Th17 and factors related to the urban environment that induce the inflammatory processes. Clinically, the serum levels of IL-17A were also associated with a poor prognosis among the patients diagnosed with MN (more thromboembolic complications and more relapses occurred) [223].

#### 3.2.2. Nephropathy IgA (IgAN)

IgAN is the most common primary glomerulonephritis in the world, with a variety of clinical symptoms characterized by recurrent hematuria or microscopic hematuria. IgAN is a group of clinicopathological syndromes with some immunopathological features in common, and many mechanisms are involved in its pathogenesis, including immunological, genetic, environmental and nutritional factors. [224]. In the case of IgAN, two interleukins deserve a special attention: IL-1 and IL-17. Patients with IgAN show an increased level of IL-17 in the serum compared to healthy people; moreover, the stimulation of human mesangial cells with IgA1 induces the production of IL-17. Additionally, IL-17 may participate in the development of nephropathy by inducing the production and glycosylation of IgA1 in B cells and may also stimulate the release of cytokines from PBMC in patients with IgAN [225]. On the other hand, IL-1 plays an important role in the progression of IgAN due to its involvement in the proliferation of mesangial cells and the production of the extracellular matrix. Additionally, the tubulointerstitial expression of IL-1*β* is significantly correlated with the rate of IgAN progression, as determined by the severity of tubulointerstitial inflammation. It was also found that patients with IgAN have a lower level of urinary IL-1Ra secretion, and patients with a higher urinary IL-1Ra/IL-1*β* ratio demonstrate a much milder histopathological picture based on renal biopsy. These data show the importance of the IL-1 cluster cytokines in the pathogenesis of IgAN [226].

The literature data indicated one more interleukin influencing the progression of IgAN, which is IL-6 [227]. Studies by Harada et al. have shown that urinary IL-6 excretion in IgAN patients is closely related to disease progression, as documented by data collected over 8 years of careful analysis. The progression of IgAN is gradual, which is why it seemed so important to conduct research aimed at capturing the clinical indicator that shows a gradual loss of kidney function and, thus, disease progression. According to the Harada report, the long-term clinical significance of IL-6 concentration in urine may not only play a diagnostic role in the emergence of primary glomerulonephritis but also predict the loss of renal function. Their multivariate analysis showed that the excretion of higher concentrations of IL-6 in urine resulted in a 7.8-fold higher risk of the worsening of renal function in patients with IgAN. The researchers also suggested that traditional prognostic factors such as hypertension and proteinuria are much weaker predictors of IgAN progression. As convinced by most of the research, it is extremely important to conduct further research aimed at determining the exact role of IL-6 in the clinical course of IgAN [228].

#### 3.2.3. Focal Segmental Glomerulosclerosis (FSGS)

FSGS is diagnosed on the basis of the histological picture, which shows segmental scarring, that covers part of the glomeruli and affects only some of the glomeruli in the collected material. Patients diagnosed with FSGS may show clinical signs of nephrotic syndrome, hematuria, hypertension or renal failure [229]. In the case of FSGS, interleukins such as IL-17, IL-6 or IL-10 may play an important role. The circulating Th17 lymphocytes assessed in peripheral blood mononuclear cells (PBMCs) were more numerous in patients with nephrotic syndrome compared to the control group. The role of the Th17/interleukin-17 (IL-17) axis was further confirmed by the finding that IL-17 staining was the most abundant in FSGS biopsies compared to MCD and mesangial proliferative glomerulonephritis. In addition, in vitro studies have demonstrated the time-dependent and dose-dependent proapoptotic effects of IL-17 on podocytes [230].

Other interleukins involved in the pathogenesis of FSGS indicated in the literature are IL-1*β*, IL-2 and IL-4. However, information on their roles in pathogenesis is scarce and requires further intensive research. In the case of IL-1*β*, Wag et al. showed its higher expression in kidney biopsy samples from FSGS patients than in patients with other types of glomerulonephritis, including minimal change disease or mesangial proliferative glomerulonephritis [231]. Studies conducted by Kalavrizioti et al. showed that the urinary excretion of IL-2, IL-4, IL-6 and IL-10 is significantly higher in patients with FSGS (also in MDC) than in the control group [232].

#### 3.2.4. Diabetic Nephropathy (DN)

DN or diabetic kidney disease is a syndrome characterized by the excretion of excess albumin in the urine, diabetic glomerular changes and a reduction in the glomerular filtration rate (GFR) in diabetics [233]. In this disease, there is an association between the circulating interleukin-10 (IL-10) levels and the degree of albuminuria in patients with DN in the development of type 1 diabetes (DM). Researchers observed significantly increased levels of circulating IL-10 in 30/30 patients with DM with DN (mean 140 pg/mL ± 102) compared with DM patients without DN, in which IL-10 was detectable in only 11/30 patients (0.79 pg/mL ± 1.24), and a group of healthy subjects, in which IL-10 was detectable only in 3/30 donors (0.92 pg/mL ± 0.17). IL-10 may be the most significant predictor of albuminuria, as a positive correlation has been demonstrated between the IL-10 values and albuminuria in patients with DM and DN. In addition, the excessive production of IL-10 may indirectly contribute to the progression of DN, which may explain the extended course of DN [234].

IL-18 may be another cytokine involved in the development of DN. This interleukin is a strong proinflammatory cytokine responsible for the release of IFN-*γ*, stimulation of the expression of a functional chemokine receptor in human mesangial cells, the synthesis of IL-1 and TNF-*α*, an increase in ICAM-1 expression and the apoptosis of endothelial cells [235]. The increased expression of IL-18 in DN patients is higher in renal tubular cells. However, the role of IL-18 is ambiguous, as many other cells can also produce this cytokine. Endothelial, epithelial, mesangial and tubular cells are not only capable of synthesizing IL-18; they may also be responsible for the production of TNF-*α*, IL-1 and IL-6, which act in a paracrine or autocrine manner [235]. The literature data also suggest the involvement of IL-6 in the development of DN. Researchers have observed higher levels of this interleukin in patients diagnosed with DN compared to diabetic patients who did not develop nephropathy [236]. This was also confirmed by other literature reports, which indicated that the functional and structural abnormalities in the course of DN and the progression of kidney damage are related to IL-6 [237,238,239,240]. Additionally, in the case of analyses carried out in animal models (rats) with diabetes, in whom the level of IL-6 mRNA in the renal cortex was determined, a positive correlation was shown related to the concentration of this cytokine in urine [235].

### 3.3. Kidney Transplantation

Kidney transplantation is the ultimate treatment for CKD. It improves the patient’s quality of life by eliminating the need for repetitive dialysis [241]. In the case of transplants, the role of IL-18 is suggested, according to the literature. When tested in animal models, caspase-1-deficient mice were protected from AKI induced by an injection of IL-18. Additionally, increased levels of IL-18 may be one of the causes of caspase-1-mediated renal ischemia [242]. In other analyses, researchers showed that higher levels of IL-18 expression in the kidney tissue of mice after 24 h correlated with disorders such as renal dysfunction while also showing elevated serum creatinine levels [242]. In addition, in transplant patients, the urinary IL-18 levels have been shown to be an early noninvasive predictor of both the need for dialysis in the first week after transplant and restoration of the transplant function after 3 months [242]. When IL-18 was measured by biopsy in patients who underwent acute rejection, strong immunoreactivity in the proximal tubules and infiltrating leukocytes into the endothelium were found. Serum IL-18 in patients with acute graft rejection compared to stable allograft recipients may lead to the conclusion that IL-18 is responsible for acute rejection; the results obtained in the studies are still inconclusive, suggesting that further research is required [242].

### 3.4. Lupus Nephritis (LN)

LN occurs in ~50% of patients with systemic lupus nephritis (SLE) and is a common, but not the only, cause of kidney injury in SLE. Men have also been found to develop the disease more aggressively (with higher rates of kidney and cardiovascular disease) and are more likely to develop renal failure than women. In addition, people who develop LN are of a younger age than patients with SLE without nephritis. It also seems important to know that LN develops early in the disease, usually within the first 6–36 months, and may be present at the initial diagnosis [243]. As with other renal diseases, immune dysregulation—in particular, cytokine imbalance—plays a key role in many human renal diseases, including LN. IL-18-binding protein (IL-18BP) is a circulating protein with a high affinity for IL-18 and can neutralize its biological activity, known as a natural IL-18 antagonist. Moreover, the plasma levels of IL-18 are elevated in LN patients compared to the group with primary nephrotic syndrome (PNS) with normal renal function. In addition, there is also an increase in the plasma IL-18BP levels in many human diseases, including acute and chronic renal failure; however, the levels of IL-18BP secretion in LN require further analysis [244].

### 3.5. Prospects of Interleukin Research in Kidney Diseases

As indicated in our review, interleukins are important in maintaining immune homeostasis and are also particularly involved in the progression of kidney disease, including nephropathy. One of the most common interleukins recurring in selected nephropathies are the proinflammatory interleukins IL-6 and IL-18. Both interleukins and their higher levels are common to each of the types of nephropathy discussed in this review. IL-6 is involved in the development of IgAN, FSMG, DN and CKD, while IL-18 is more frequently studied in LN, DN and AKI [192,196,197,198,207,210,228,235,242,244]. On this basis, it can be hypothesized that both interleukins could be additional biomarkers facilitating differentiation into the selected type of nephropathy. In contrast to proinflammatory interleukins are anti-inflammatory interleukins. The most frequently mentioned in scientific studies on nephropathy was IL-10, occurring, inter alia, in DN, FSMGS, MN, AKI and IL-20 found in CKD and AKI [209,210,215,216,217,218,233,234]. This may lead to the hypothesis that, at the stage of development of individual nephropathies, we are dealing with a higher level of IL-10, while, in the case of acute or chronic inflammation of the kidneys, which may develop as a result of primary diseases, we have more studies confirming the presence of IL-20. Such knowledge may indicate the potential role of the above-mentioned interleukins as prognostic factors related to the development of kidney diseases, as well as its transition into a chronic state. In addition, the action of proinflammatory interleukins may be one of the factors influencing the disease progression and, consequently, the patient’s prognosis. Therefore, another important challenge for future research would be to verify the potential role of proinflammatory interleukins (IL-4, IL-6, IL-17 and IL-18). in the diagnosis process, their importance in disease progression in correlation with the other morphological and immunological parameters of patients. It also seems important to try to verify in an animal model whether interleukin-targeted therapy in selected types of nephropathy would have a positive effect on the prognosis, progression and treatment of kidney diseases. Additional interleukins that, based on the literature data presented in the review, may be involved in the progression of glomerulopathy are IL-1 and IL-2 [226,232]. In light of the above information, and due to the variety of functions they perform in the immune system (maintaining the immune homeostasis), as well as their importance in pathological conditions, interleukins constitute an important research material. However, their importance in the progression of kidney disease is still not fully understood and requires further interdisciplinary research involving animal models and larger groups of patients.

## 4. Materials and Methods

### 4.1. Bioinformatic Analyses of the Amino Acid Sequences of Interleukins

For bioinformatic analyses, the amino acid sequences deposited in the UniProt database [245] were used. The interleukin identification numbers and their amino acid sequences are provided in Supplementary Materials Table S1. These sequences were used to carry out further bioinformatics analyses. The sequence length and molecular weight of individual interleukins were taken from the UniProt database. The determination of the isoelectric point of the tested interleukins and their amino acid compositions was carried out using the IPC isoelectric point calculator software available online [246]. The analysis of the second-order structure of the interleukins was carried out using the NetSurfP-2.0 program available online [247].

### 4.2. Analysis of the Amino Acid Sequence Identity of Interleukins

The amino acid sequences of the selected interleukins from the UniProt databases (Supplementary Materials Table S1) were used for the analyses. All interleukins IL-1–IL-40 were analyzed, except for IL-16, which is pro-IL-16, and the dimeric interleukins IL-35 and IL-39. The amino acid sequences of individual interleukins were compared with each other using the Clustal Omega program available at Reference [248]. The results of the analyses were presented as the percentage of identical amino acids in the analyzed amino acid sequences and collected in the Supplementary Materials—Table S2.

## 5. Conclusions

Due to the increasing number of patients diagnosed with renal dysfunction leading to the development of serious diseases, it is extremely important to explain the mechanisms of their formation and progression. This is undoubtedly one of the greatest challenges for modern medicine. That is why many researchers, scientists and doctors create interdisciplinary multilevel teams aimed at explaining a small fragment of the etiopathogenesis of kidney diseases, which concerns the role of the immune system in the development of these disease entities. Their joint efforts result in the progress of new trends, diagnostic tests, methods of care and therapy of patients in whom, as a result of a variety of genetic, environmental or lifestyle factors, there is a sudden loss or deterioration of kidney function. Only thanks to a comprehensive approach of many specialists was it possible to demonstrate the important role of interleukins in the development and progression of several kidney diseases classified as AKI or CKD and kidney transplantation. Due to the pleiotropic mechanisms of action and the number of interleukins in the human body, it is extremely difficult to establish the unequivocal effects of individual cytokines on the development of individual disease entities. That is why it is so important to realize the role of interleukins in maintaining the homeostasis of the immune system and cellular processes taking place in the human body and all the consequences of their disorders that determine the emergence and progression of disease states.

## Figures and Tables

**Table 1:** Molecular characteristics of the interleukins.

Name	Number of Amino Acids	Molecular Mass [kDa]	Isoelectric Point	Percentage of Hydrophilic Amino Acids	Percentage of Hydrophobic Amino Acids	Secondary Structure		Receptors	Reference
						*α*-Helix	Β-Strand		
IL-1	219	24.243	6.55	59.82	40.18	1	12	IL-1R1, IL-1R2	[46]
IL-2	153	17.628	7.08	56.21	43.79	5	3	IL-2RA, IL-2RB, IL-2RG	[47]
IL-3	152	17.233	7.96	50.66	49.34	5	1	IL-3RA, IL-3RB	[48]
IL-4	153	17.492	8.15	62.74	37.26	7	2	IL-4R	[49]
IL-5	134	15.238	7.44	56.72	43.28	4	0	IL-5RA, IL-3RB	[50]
IL-6	212	23.718	5.98	56.60	43.40	6	0	IL-6R	[51]
IL-7	177	20.187	7.83	59.32	40.68	4	0	IL-7R	[52]
IL-8	99	11.098	8.11	53.53	46.47	2	4	IL8RB	[53]
IL-9	144	15.909	7.72	59.03	40.97	5	0	IL-9R	[54]
IL-10	178	20.517	7.23	59.55	40.45	4	0	IL-10RA	[55]
IL-11	199	21.429	10.5	44.72	55.28	7	0	IL-11RA	[56]
IL-12 subunit alpha	219	24.874	5.93	55.25	44.75	5	0	IL-12RB1	[57]
IL-12 subunit beta	328	37.169	5.36	63.11	36.89	2	24		[58]
IL-13	146	15.816	7.64	49.31	50.69	4	1	IL-13RA1, IL-13RA2	[59]
IL-14	546	61.891	5.93	69.05	30.95	2	0	Unkown	[60]
IL-15	162	18.086	5.00	58.02	41.96	5	0	IL-15RA	[61]
Pro-IL-16	1332	141.752	7.19	61.94	38.06	10	24	CD4	[62]
IL-17A	155	17.504	7.76	59.35	40.65	1	6	IL-17RA	[63]
IL-18	193	22.326	4.41	61.66	38.34	2	16	IL-18R1	[64]
IL-19	177	20.452	6.88	57.63	42.37	7	0	IL-20R	[65]
IL-20	176	20.072	7.91	59.09	40.91	6	1	IL-20R	[66]
IL-21	162	18.653	8.67	63.58	36.42	6	0	IL-21R	[67]
IL-22	179	20.011	7.00	55.31	44.69	7	0	IL2-2RA1	[68]
IL-23 alfa	189	20.730	5.73	53.97	46.03	7	0	IL-23R	[69]
IL-24	206	23.825	8.05	51.94	48.06	6	1	IL-20R	[70]
IL-25	177	20.330	7.55	62.15	37.85	0	6	LY6E	[71]
IL-26	171	19.843	9.22	57.89	42.11	5	0	IL-20R1	[72]
IL-27 subunit alpha	243	27.493	5.94	51.44	48.56	7	0	IL-27RA	[73]
IL-27 subunit beta	229	25.396	8.83	49.78	50.22	1	15	IL-27RA	[74]
IL-28 Interferon lambda receptor 1	520	57.653	4.8	57.11	42.89	3	19	IL-28R	[75]
IL-29 Interferon lambda-1	200	21.898	8.09	52.50	47.50	8	0	Unknown	[76]
IL-30	243	27.493	5.94	51.44	48.56	7	0	Unknown	[77]
IL-31	164	18.205	5.12	57.32	42.68	4	0	IL-31RA	[77,78]
IL-32	234	26.676	5.03	58.97	41.03	8	2	Unknown	[77,79]
IL-33	270	30.759	7.86	64.81	35.19	4	15	Unknown	[77,80]
IL-34	242	27.482	6.47	54.96	45.04	7	1	Unknown	[77,81]
IL-35	it consists of two subunits: IL-12α and IL-27β							Unknown	[77]
IL-36 alpha	158	17.684	5.65	56.96	43.04	2	12	Unknown	[77,82]
IL-36 beta	164	18.522	8.72	62.19	37.81	0	10	Unknown	[77,83]
IL-36 gamma	168	18.721	4.94	59.76	40.24	2	13	Unknown	[77,84]
IL-37	218	24.126	5.82	59.17	40.83	1	12	IL-18Ra, IL-18BP	[77,85]
IL-38 Interleukin-1 family member 10	152	16.943	4.88	57.89	42.11	2	12	IL-1R1, IL-36R	[77,86]
IL-39	composed of the IL-23p19 alpha subunit and Ebi3 beta subunit.							IL-23R, IL-27R, and gp130	[45,77]
IL-40	265	29.091	7.83	54.72	45.28	1	18		[77,87]

**Table 2:** Influence of mutations of selected interleukins on their activity.

Name	Mutation Site	Change	Effect	Reference
IL-6	173	A → V	Almost no loss of activity	[51,88]
	185	W → R	No loss of activity	
	204	S → P	87% loss of activity	
	210	R → K, E, Q, T, A or P	Loss of activity	
	212	M → T, N, S or R		
IL-17A	69	R → A	Impairs binding to IL-17RA and IL-17RC	[63,89]
	78	R → V	Decreases the affinity for IL-17RA by 5-fold	[63,90]
	90	W → V	Has no effect on the affinity for IL-17RA	
	108	Y → I	Decreases the affinity for IL-17RA	
	109	H → S		
IL-18	40	K → A	Reduces binding to IL-18R1 and the ability to induce IFNG production	[64,91]
	41	L → A	Impairs binding to IL-18R1 and the ability to induce IFNG production	
	44	K → A:	Reduces binding to IL-18R1 and the ability to induce IFNG production	
	49	R → A		
	53	D → A		
	69	M → A	Impairs binding to IL-18R1 and the ability to induce IFNG production	
	71	D → A		
	94	R → A		
	96	M → A		
	115	K → A	Reduces binding of the preformed binary complex of IL-18 and IL-18R1 to IL-18RAP resulting in impaired IFNG production	
	120	K → A		
	134	D → A		
	140	R → A	Reduces binding to IL-18R1 and the ability to induce IFNG production	
	144	G → A	Abolishes binding of the preformed binary complex of IL-18 and IL-18R1 to IL-18RAP	
	145	H → A		
	146	D → A	Reduces binding of the preformed binary complex of IL-18 and IL-18R1 to IL-18RAP	
	148	K → A	Abolishes binding of the preformed binary complex of IL-18 and IL-18R1 to IL-18RAP	
	168	D → A	Reduces binding to IL-18R1 and the ability to induce IFNG production	
	183	R → A	Reduces binding of the preformed binary complex of IL-18 and IL-18R1 to IL-18RAP	
	186	M → A		
IL-33	144	E → K	Decreases affinity for IL-1RL1	[80,92]
	148	E → K	7-fold decrease in affinity for IL-1RL1	
	149	D → K	Almost abolishes binding to IL-1RL1	
	165	E → K	8-fold decrease in affinity for IL-1RL1	
	244	D → K	Decreases affinity for IL-1RL1	

**Table 3:** Origin and functions of human interleukins.

Name	Origin/Source	Target Cells	Functions	Link to Disease	Reference
IL-1	Monocytes, macrophages, lymphocytes, neutrophils, fibroblasts	NK cells, Th cells, B cells	Lymphocyte activation, fever, regulates sleep, proinflamatory cytokine, maturation and proliferation	Inflamatory diseases, Autoimmune diseases	[93,94]
IL-2	Th1 cells	T cells, B cells, macrophages	Stimulates growth of T cells	Autoimmune diseases (T cel-mediated)	[95,96,97]
IL-3	Th cells and mast cells	Mast cells, hemapoetic stem cells	Stimulates bone marrow growth	Cancers, allergic diseases	[98,99]
IL-4	Th2 cells, basophils, NKT cells	T cells, B cells	B-cell growth factor, role in tissue adhesion and inflamation	Autoimmune diseases, CLL	[100,101]
IL-5	T cells	Mast cells. Eosinophils	Activated T cells, Differentiation and function of myeloid cells	Asthma, Allergy	[102,103]
IL-6	Monocytes, macrophages,	hemapoetic cells	Activated T cells, contributes to host defense through the stimulation of acute phase responses, hematopoiesis	Autoimmune diseases, multiple myeloma	[104,105]
IL-7	Monocytes, macrophages, epithelial cells	T cells, B cells, NK cells	T-cell development, survival and homeostasis of mature T cells, B cells and T-cell proliferation	Allergy	[106,107]
IL-8	Monocytes and fibroblasts	Neutrophils, eosinofhils, basophils, endothelial cells, keratinocytes	Angiogenesis, induces chemotaxis, stimulates phagocytosis, neutrophil chemotaxis, superoxide release and granule release	Inflamatory diseases	[108,109]
IL-9	Eosinophils, mast cells	T cells, B cells, mast cells	Chemokine, Mast and T-cell growth factor and enhances T-cell survival, mast cell activation and synergy with erythropoietin	Asthma, food allergy, Hodgin’s	[110,111]
IL-10	Macrophages, T cells, B cells, dendritic cells	Mocnocytes, macrophages	Immune supressed	Cancer, allergic reaction	[112,113]
IL-11	Bone marrow, stromal cells	Hepatocyte, myeloid	Synergistic effect on hematopoesis, growth factor for myeloid, osteoclast formation, colony stimulating factor, raised platelet count in vivo and inhibition of proinflammatory cytokine production	allergic reaction	[114,115]
IL-12	stromal cells, macrophages, B cells	T cells, myeloid	Proinflammatory cytokine that regulates T-cell and natural killer cell responses, induces the production of interferon-*γ*, growth factor for myeloid and induction of Th1 cells	allergic reaction	[116,117]
IL-13	CD4+ T cells (Th2), NKT cells and mast cells	monocytes, fibroblasts, epithelial cells and B cells	Growth factor for myeloid, B-cell growth and differentiation, stimulates isotype switching to IgE, increased collagen synthesis by fibroblasts and inhibits proinflammatory cytokine production	allergic reaction, asthma	[118,119]
IL-14	T cells	B cells	Activated B-cell proliferation and inhibition of immunoglobulin secretion	allergic reaction	[120]
IL-15	Monocytes, epithelium, and muscles	T cells and activated B cells	Proliferation of both B and T cells	Autoimmune diseases	[121,122]
Pro-IL-16	Eosinophils and CD8+ T cells	CD4+ T cells	CD4+ T cell chemoattraction	Infectious diseases	[123]
IL-17A	Th-17, NK cells, neutrophils	epithelial and endothelial cells, monocytes, macrophages	Release of IL-6 and other proinflammatory cytokines; stimulates chemokine synthesis by endothelial cells	Contact hypersensitivity, atopic dermatitis	[124,125]
IL-18	Macrophages, osteoblast, dendritic cells	T cells, NK cells	Causes interferon gamma production and enhances NK cell activity	Autoimmune diseases, psorasis	[126,127]
IL-19	Th2 lymphocytes, monocytes,	macrophages, T cells, B cells, endothelial cells and brain resident glial cells	An anti-inflammatory molecule. It promotes immune responses mediated by regulatory lymphocytes	psorasis	[128,129]
IL-20	immune cells and activated epithelial cells	Keratinocytes, monocytes	Skin biology, cellular communication between epithelial cells and the immune system under inflammatory conditions	Psorasis, RA	[130,131]
IL-21	NK cells, CD4+ T cells	T cells, B cells, dendritic cells, macrophages, keratinocytes	Promotes B- and T-lymphocyte proliferation and differentiation	Cancer, SLE Parasitic diseases, RA	[132,133,134]
IL-22	Activated T cells	Tissue cells, keratinocytes	Inhibits IL-4 production; mucosal surface protection and tissue repair	Psorasis, cancer, IBD	[135,136,137]
IL-23	Macrophages, dendritc cells	T cells, Macrophages	IL-17-producing T cells, promote memory T-cell proliferation	sensitivity to external pathogens	[138,139]
IL-24	Monocytes, T and B cells	Cancer cells	Cancer-specific cell death, causes wound healing and protects against bacterial infections and cardiovascular diseases	Melanoma, psorasis	[140,141,142]
IL-25	Dendritic cells	various types of cells, including Th2 cells	Stimulates the synthesis of the Th2 cytokine profile, including IL-4 and IL-13	Asthma, autoimmune diseases	[143,144]
IL-26	Activated T cells, NK cells	epithelial cells and intestinal epithelial cells	Induces IL-10 expression; stimulates the production of IL-1-beta, IL-6 and IL-8 and causes Th17 cell generation	IBD	[145,146]
IL-27	T cells, activated dendritic cells	T cells, NK cells	Stimulates IL-10 production, upregulates type-2 interferon synthesis by natural killer cells	Immune pathology	[147,148,149]
IL-28 Interferon lambda-1	Regulatory T-cells	keratinocytes and melanocytes	Role in immune defense against viruses, upregulates TLR-2 and TLR-3 expression. IL-28 enhances the keratinocyte capacity to recognize pathogens in the healthy skin	Allergic reaction	[150,151]
IL-29 Interferon lambda-1	dendritic cells, and regulatory T cells	Tissue cells	Viral protective responses	Allergic reaction, cancer	[150,152]
IL-30	Monocytes	monocytes, macrophages, dendritic cells, T and B lymphocytes, natural killer cells, mast cells, and endothelial cell	Regulate inflammation by inhibiting Th17 cells production using the STAT1 pathway	Cancer, psorasis	[153,154]
IL-31	Th2 cells and dendritic cells	Monocytes, basophils, keratinocytes	Induces chemokines production and synthesis of IL-6, IL-16 and IL-32, helps trigger cell-mediated immunity against pathogens	Autoimmune skin diseases	[155,156]
IL-32	Monocytes, NK cells	Monocytes, macrophages	Induces the synthesis of various cytokines including IL-6 and IL-1beta. It inhibits IL-15 production	Asthma, cancer	[157,158]
IL-33	Mast cells and Th2 lymphocytes	dendritic cells and T and B lymphocytes	Induces helper T cells, mast cells, eosinophils and basophils to produce type 2 cytokines, protection against parasites and type-I hypersensitivity reaction	Dermatitis, allergy, infectious and inflammatory diseases	[159,160]
IL-34	Heart, colon, prostate	Monocytes, macrophages	Enhances IL-6 production and participates in the differentiation and development of antigen-presenting cells, including microglia	RA, artritis	[161,162,163]
IL-35	B cells	NK cells, activated T cells	Immune suppression, involvement in lymphocyte differentiation	RA, artritis	[164,165]
IL-36	Tissue cells, skin cells	T lymphocytes and NK cells	Regulating the IFN-*γ* synthesis, stimulates the hematopoiesis and expression of both MHC class I and II molecules	Immune responsce, inflamatory diseases	[166,167]
IL-37	monocytes	Dendritic cells	Regulation of the innate immunity causing immunosuppression	autoimmune disorders	[168,169]
IL-38 Interleukin-1 family member 10	placenta, heart, and brain, tonsils B cells, spleen, skin, and thymus	T cells	Inhibits the synthesis of IL-17 and IL-22	Inflamatory diseases	[170,171]
IL-39	B cells	neutrophils	Neutrophils differentiation or expansion	systemic lupus erythematosus, acute coronary syndrome	[45]
IL-40	bone marrow, fetal liver, and by activated B cells	B cells	Development of humoral immune responses, involved in IgA production and B cell homeostasis and development	Lymphoma	[77,172]

## Data Availability

Not applicable.

## Supplementary Materials

The following are available online at https://www.mdpi.com/article/10.3390/ijms23020647/s1.
