# Supplementary material for: Biological Role, Mechanism of Action and the Importance of Interleukins in Kidney Diseases
Source: Int J Mol Sci. 2022 Jan 7;23(2):647. doi: 10.3390/ijms23020647 (PMC8775480; doi:10.3390/ijms23020647)
Supplement: Supplementary file 1 [file ijms-23-00647-s001.zip › Supplementary Materials-Table S1.pdf]

**Supplementary Materials:**

**Table S1.** Protein ID and amino acid sequences of interleukins used for bioinformatics analyzes

| Name | Protein ID | Sequence |
|---|---|---|
| **IL-1 $\alpha$** | **Q7RU00** | MSFVGENSGVKMGSEDWEKDEPQCCLEDPAGSPLEPGPSLPTMNFVHTSRKVKSLNPKKF<br>SIHDQDHKVLVLDSGNLIAVPDKNYIRPEIFFALASSLSSASAEKGSLILLGVSKGEFCL<br>YCDKDKGQSHPSLQLKKEKLMKLAAQKESARRPFIFYRAQVGSRNMLESAAHPGWFICTS<br>CNCNEPVGVTDKFENRKHIEFSFQPVVCKAEMSPSEVSN |
| **IL-2** | P60568 | MYRMQLLSCIALSLALVTNSAPTSSSTKKTQLQLEHLLLDLQMILNGINNYKNPKLTRML<br>TFKFYMPKKATELKHLQCLEEELKPLEEVLNLAQSKNFHLRPRDLISNINVIVLELKGSE<br>TTFMCEYADETATIVEFLNRWITFCQSIISTLT |
| **IL-3** | P08700 | MSRLPVLLLLQLLVRPGLQAPMTQTTPLKTSWVNCSNMIDEIITHLKQPPLPLLDFNNLN<br>GEDQDILMENNLRRPNLEAFNRAVKSLQNASAIESILKNLLPCLPLATAAPTRHPIHIKD<br>GDWNEFRRKLTFYLKTLENAQAQQTTLSLAIF |
| **IL-4** | P05112 | MGLTSQLLPPLFFLLACAGNFVHGHKCDITLQEIIKTLNSLTEQKTLCTELTVTDIFAAS<br>KNTTEKETFCRAATVLRQFYSHHEKDTRCLGATAQQFHRHKQLIRFLKRLDRNLWGLAGL<br>NSCPVKEANQSTLENFLERLKTIMREKYSKCSS |
| **IL-5** | P05113 | MRMLLHLSLLALGAAYVYAIPTEIPTSALVKETLALLSTHRTLLIANETLRIPVPVHKNH<br>QLCTEEIFQGIGTLESQTVQGGTVERLFKNLSLIKKYIDGQKKKCGEERRRVNQFLDYLQ<br>EFLGVMNTEWIIES |
| **IL-6** | P05231 | MNSFSTSAFGPVAFSLGLLLVLPAAFPAPVPPGEDSKDVAAPHRQPLTSSERIDKQIRYI<br>LDGISALRKETCNKSNMCESSKEALAENNLNLPKMAEKDGCFQSGFNEETCLVKIITGLL<br>EFEVYLEYLQNRFESSEEQARAVQMSTKVLIQFLQKKAKNLDAITTPDPTTNASLLTKLQ<br>AQNQWLQDMTTHLILRSFKEFLQSSLRALRQM |
| **IL-7** | P13232 | MFHVSFRYIFGLPPLILVLLPVASSDCDIEGKDGKQYESVLMVSIDQLLDSMKEIGSNCL<br>NNEFNFFKRHICDANKEGMFLFRAARKLRQFLKMNSTGDFDLHLLKVSEGTTILLNCTGQ<br>VKGRKPAALGEAQPTKSLEENKSLKEQKKLNDLCFLKRLLQEIKTCWNKILMGTKEH |
| **IL-8** | P10145 | MTSKLAVALLAAFLISAALCEGAVLPRSAKELRCQCIKTYSKPFHPKFIKELRVIESGPH<br>CANTEIIVKLSDGRELCLDPKENWVQRVVEKFLKRAENS |
| **IL-9** | P15248 | MLLAMVLTSALLLCSVAGQGCPTLAGILDINFLINKMQEDPASKCHCSANVTSCLCLGIP<br>SDNCTRPCFSERLSQMTNTTMQTRYPLIFSRVKKSVEVLKNNKCPYFSCEQPCNQTTAGN<br>ALTFLKSLLEIFQKEKMRGMRGKI |
| **IL-10** | P22301 | MHSSALLCCLVLLTGVRASPGQGTQSENSCTHFPGNLPNMLRDLRDAFSRVKTFFQMKDQ<br>LDNLLLKESLLEDFKGYLGCQALSEMIQFYLEEVMPQAENQDPDIKAHVNSLGENLKTLR<br>LRLRRCHRFLPCENKSKAVEQVKNAFNKLQEKGIYKAMSEFDIFINYIEAYMTMKIRN |
| **IL-11** | P20809 | MNCVCRLVLVVLSLWPDTAVAPGPPPGPPRVSPDPRAELDSTVLLTRSLLADTRQLAAQL<br>RDKFPADGDHNLDSLPTLAMSAGALGALQLPGVLTRLRADLLSYLRHVQWLRRAGGSSLK<br>TLEPELGTLQARLDRLLRRLQLLMSRLALPQPPPDPPAPPLAPPSSAWGGIRAAHAILGG<br>LHLTLDWAVRGLLLLKTRL |
| **IL-12 $\alpha$** | P29459 | MCPARSLLLVATLVLLDHLSLARNLPVATPDPGMFPCLHHSQNLLRAVSNMLQKARQTLE<br>FYPCTSEEIDHEDITKDKTSTVEACLPLELTKNESCLNSRETSFITNGSCLASRKTSFMM<br>ALCLSSIYEDLKMYQVEFKTMNAKLLMDPKRQIFLDQNMLAVIDELMQALNFNSETVPQK<br>SSLEEPDFYKTKIKLCILLHAFRIRAVTIDRVMSYLNAS |
| **IL-12 $\beta$** | P29460 | MCHQQLVISWFSLVFLASPLVAIWELKKDVYVVELDWYPDAPGEMVVLTCDTPEEDGITW<br>TLDQSSEVLGSGKTLTIQVKEFGDAGQYTCHKGGEVLSHSLLLLHKKEDGIWSTDILKDQ<br>KEPKNKTFLRCEAKNYSGRFTCWWLTTISTDLTFSVKSSRGSSDPQGVTCGAATLSAERV<br>RGDNKEYEYSVECQEDSACPAAEESLPIEVMVDAVHKLKYENYTSSFFIRDIIKPDPPKN<br>LQLKPLKNSRQVEVSWEYPDTWSTPHSYFSLTFCVQVQGKSKREKKDRVFTDKTSATVIC<br>RKNASISVRAQDRYYSSSWSEWASVPCS |
| **IL-13** | P35225 | MHPLLNPLLLALGLMALLLTTVIALTCLGGFASPGPVPPSTALRELIEELVNITQNQKAP<br>LCNGSMVWSINLTAGMYCAALESLINVSGCSAIEKTQRMLSGFCPHKVSAGQFSSLHVRD<br>TKIEVAQFVKDLLLHLKKLFREGRFN |
| **IL-14/TXLNA** | P40222 | MKNQDKKNGAAKQSNPKSSPGQPEAGPEGAQERPSQAAPAVEAEGPGSSQAPRKPEGAQA<br>RTAQSGALRDVSEELSRQLEDILSTYCVDNNQGGPGEDGAQGEPAEPEDAEKSRTYVARN |

| | | |
|---|---|---|
| | | GEPEPTPVVNGEKEPSKGDPNTEEIRQSDEVGDRDHRRPQEKKKAKGLGKEITLLMQTLN<br>TLSTPEEKLAALCKKYAELLEEHRNSQKQMKLLQKKQSQLVQEKDHLRGEHSKAVLARSK<br>LESLCRELQRHNRSLKEEGVQRAREEEEKRKEVTSHFQVTLNDIQLQMEQHNERNSKLRQ<br>ENMELAERLKKLIEQYELREEHIDKVFKHKDLQQQLVDAKLQQAQEMLKEAEERHQREKD<br>FLLKEAVESQRMCELMKQQETHLKQQLALYTEKFEEFQNTLSKSSEVFTTFKQEMEKMTK<br>KIKKLEKETTMYRSRWESSNKALLEMAEEKTVRDKELEGLQVKIQRLEKLCRALQTERND<br>LNKRVQDLSAGGQGSLTDSGPERRPEGPGAQAPSSPRVTEAPCYPGAPSTEASGQTGPQE<br>PTSARA |
| **IL-15** | P40933 | MRISKPHLRSISIQCYLCLLLNSHFLTEAGIHVFILGCFSAGLPKTEANWVNVISDLKKI<br>EDLIQSMHIDATLYTESDVHPSCKVTAMKCFLLELQVISLESGDASIHDTVENLIILANN<br>SLSSNGNVTESGCKECEELEEKNIKEFLQSFVHIVQMFINTS |
| **IL-16** | Q14005 | MESHSRAGKSRKSAKFRSISRSLMLCNAKTSDDGSSPDEKYPDPFEISLAQGKEGIFHSS<br>VQLADTSEAGPSSVPDLALASEAAQLQAAGNDRGKTCRRIFFMKESSTASSREKPGKLEA<br>QSSNFLFPKACHQRARSNSTSVNPYCTREIDFPMTKKSAAPTDRQPYSLCSNRKSLSQQL<br>DCPAGKAAGTSRPTRSLSTAQLVQPSGGLQASVISNIVLMKGQAKGLGFSIVGGKDSIYG<br>PIGIYVKTIFAGGAAAADGRLQEGDEILELNGESMAGLTHQDALQKFKQAKKGLLTLTVR<br>TRLTAPPSLCSHLSPPLCRSLSSSTCITKDSSSFALESPSAPISTAKPNYRIMVEVSLQK<br>EAGVGLGIGLCSVPYFQCISGIFVHTLSPGSVAHLDGRLRCGDEIVEISDSPVHCLTLNE<br>VYTILSHCDPGPVPIIVSRHPDPQVSEQQLKEAVAQAVENTKFGKERHQWSLEGVKRLES<br>SWHGRPTLEKEREKNSAPPHRRAQKVMIRSSSDSSYMSGSPGGSPGSGSAEKPSSDVDIS<br>THSPSLPLAREPVVLSIASSRLPQESPPLPESRDSHPPLRLKKSFEILVRKPMSSKPKPP<br>PRKYFKSDSDPQKSLEERENSSCSSGHTPPTCGQEARELLPLLLPQEDTAGRSPSASAGC<br>PGPGIGPQTKSSTEGEPGWRRASPVTQTSPIKHPLLKRQARMDYSFDTTAEDPWVRISDC<br>IKNLFSPIMSENHGHMPLQPNASLNEEEGTQGHPDGTPPKLDTANGTPKVYKSADSSTVK<br>KGPPVAPKPAWFRQSLKGLRNRASDPRGLPDPALSTQPAPASREHLGSHIRASSSSSSIR<br>QRISSFETFGSSQLPDKGAQRLSLQPSSGEAAKPLGKHEEGRFSGLLGRGAAPTLVPQQP<br>EQVLSSGSPAASEARDPGVSESPPPGRQPNQKTLPPGPDPLLRLLSTQAEESQGPVLKMP<br>SQRARSFPLTRSQSCETKLLDEKTSKLYSISSQVSSAVMKSLLCLPSSISCAQTPCIPKE<br>GASPTSSSNEDSAANGSAETSALDTGFSLNLSELREYTEGLTEAKEDDDGDHSSLQSGQS<br>VISLLSSEELKKLIEEVKVLDEATLKQLDGIHVTILHKEEGAGLGFSLAGGADLENKVIT<br>VHRVFPNGLASQEGTIQKGNEVLSINGKSLKGTTHHDALAILRQAREPRQAVIVTRKLTP<br>EAMPDLNSSTDSAASASAASDVSVESTAEATVCTVTLEKMSAGLGFSLEGGKGSLHGDKP<br>LTINRIFKGAASEQSETVQPGDEILQLGGTAMQGLTRFEAWNIIKALPDGPVTIVIRRKS<br>LQSKETTAAGDS |
| **IL-17 $\alpha$** | Q16552 | MTPGKTSLVSLLLLLSLEAIVKAGITIPRNPGCPNSEDKNFPRTVMVNLNIHNRNTNTNP<br>KRSSDYYNRSTSPWNLHRNEDPERYPSVIWEAKCRHLGCINADGNVDYHMNSVPIQQEIL<br>VLRREPPHCPNSFRLEKILVSVGCTCVTPIVHHVA |
| **IL-18** | Q14116 | MAAEPVEDNCINFVAMKFIDNTLYFIAEDDENLESDYFGKLESKLSVIRNLNDQVLFIDQ<br>GNRPLFEDMTDSDCRDNAPRTIFIISMYKDSQPRGMAVTISVKCEKISTLSCENKIISFK<br>EMNPPDNIKDTKSDIIFFQRSVPGHDNKMQFESSSYEGYFLACEKERDLFKLILKKEDEL<br>GDRSIMFTVQNED |
| **IL-19** | Q9UHD0 | MKLQCVSLWLLGTILILCSVDNHGLRRCLISTDMHHIEESFQEIKRAIQAKDTFPNVTIL<br>STLETLQIIKPLDVCCVTKNLLAFYVDRVFKDHQEPNPKILRKISSIANSFLYMQKTLRQ<br>CQEQRQCHCRQEATNATRVIHDNYDQLEVHAAAIKSLGELDVFLAWINKNHEVMFSA |
| **IL-20** | Q9NYY1 | MKASSLAFSLLSAAFYLLWTPSTGLKTLNLGSCVIATNLQEIRNGFSEIRGSVQAKDGNI<br>DIRILRRTESLQDTKPANRCCLLRHLLRLYLDRVFKNYQTPDHYTLRKISSLANSFLTIK<br>KDLRLCHAHMTCHCGEEAMKKYSQILSHFEKLEPQAAVVKALGELDILLQWMEETE |
| **IL-21** | Q9HBE4 | MRSSPGNMERIVICLMVIFLGTLVHKSSSQGQDRHMIRMRQLIDIVDQLKNYVNDLVPEF<br>LPAPEDVETNCEWSAFSCFQKAQLKSANTGNNERIINVSIKKLKRKPPSTNAGRRQKHRL<br>TCPSCDSYEKKPPKEFLERFKSLLQKMIHQHLSSRTHGSEDS |
| **IL-22** | Q9GZX6 | MAALQKSVSSFLMGTLATSCLLLLALLVQGGAAAPISSHCRLDKSNFQQPYITNRTFMLA<br>KEASLADNNTDVRLIGEKLFHGVSMSERCYLMKQVLNFTLEEVLFPQSDRFQPYMQEVVP<br>FLARLSNRLSTCHIEGDDLHIQRNVQKLKDTVKKLGESGEIKAIGELDLLFMSLRNACI |
| **IL-23$\alpha$** | Q9NPF7 | MLGSRAVMLLLLLPWTAQGRAVPGGSSPAWTQCQQLSQKLCTLAWSAHPLVGHMDLREEG<br>DEETTNDVPHIQCGDGCDPQGLRDNSQFCLQRIHQGLIFYEKLLGSDIFTGEPSLLPDSP<br>VGQLHASLLGLSQLLQPEGHHWETQQIPSLSPSQPWQRLLLRFKILRSLQAFVAVAARVF<br>AHGAATLSP |

| | | |
|---|---|---|
| **IL-24** | Q13007 | MNFQQRLQSLWTLARPFCPPLLATASQMQMVVLPCLGFTLLLWSQVSGAQGQEFHFGPCQ VKGVVPQKLWEAFWAVKDTMQAQDNITSARLLQQEVLQNVSDAESCYLVHTLLEFYLKTV FKNYHNRTVEVRTLKSFSTLANNFVLIVSQLQPSQENEMFSIRDSAHRRFLLFRRAFKQL DVEAALTKALGEVDILLTWMQKFYKL |
| **IL-25** | Q9H293 | MRERPRLGEDSSLISLFLQVVAFLAMVMGTHTYSHWPSCCPSKGQDTSEELLRWSTVPVP PLEPARPNRHPESCRASEDGPLNSRAISPWRYELDRDLNRLPQDLYHARCLCPHCVSLQT GSHMDPRGNSELLYHNQTVFYRRPCHGEKGTHKGYCLERRLYRVSLACVCVRPRVMG |
| **IL-26** | Q9NHP9 | MLVNFILRCGLLLVTLSLAIAKHKQSSFTKSCYPRGTLSQAVDALYIKAAWLKATIPEDR IKNIRLLKKKTKKQFMKNCQFQEQLLSFFMEDVFGQLQLQGCKKIRFVEDFHSLRQKLSH CISCASSAREMKSITRMKRIFYRIGNKGIYKAISELDILLSWIKKLLESSQ |
| **IL-27 α** | Q8NEV9 | MGQTAGDLGWRLSLLLLPLLLVQAGVWGFPRPPGRPQLSLQELRREFTVSLHLARKLLSE VRGQAHRFAESHLPGVNLYLLPLGEQLPDVSLTFQAWRRLSDPERLCFISTTLQPFHALL GGLGTQGRWTNMERMQLWAMRLDLRDLQRHLRFQVLAAGFNLPEEEEEEEEEEEEERKGL LPGALGSALQGPAQVSWPQLLSTYRLLHSLELVLSRAVRELLLLSKAGHSVWPLGFPTLS PQP |
| **IL-27 β** | Q14213 | MTPQLLLALVLWASCPPCSGRKGPPAALTLPRVQCRASRYPIAVDCSWTLPPAPNSTSPV SFIATYRLGMAARGHSWPCLQQTPTSTSCTITDVQLFSMAPYVLNVTAVHPWGSSSSFVP FITEHIIKPDPPEGVRLSPLAERQLQVQWEPPGSWPFPEIFSLKYWIRYKRQGAARFHRV GPIEATSFILRAVRPRARYYVQVAAQDLTDYGELSDWSLPATATMSLGK |
| **IL-28** | Q8IU57 | MAGPERWGPLLLCLLQAAPGRPRLAPPQNVTLLSQNFSVYLTWLPGLGNPQDVTYFVAYQ SSPTRRRWREVEECAGTKELLCSMMCLKKQDLYNKFKGRVRTVSPSSKSPWVESEYLDYL FEVEPAPPVLVLTQTEEILSANATYQLPPCMPPLDLKYEVAFWKEGAGNKTLFPVTPHGQ PVQITLQPAASEHHCLSARTIYTFSVPKYSKFSKPTCFLLEVPEANWAFLVLPSLLILLL VIAAGGVIWKTLMGNPWFQRAKMPRALDFSGHTHPVATFQPSRPESVNDLFLCPQKELTR GVRPTPRVRAPATQQTRWKKDLAEDEEEEDEEDTEDGVSFQPYIEPPSFLGQEHQAPGHS EAGGVDSGRPRAPLVPSEGSSAWDSSDRSWASTVDSSWDRAGSSGYLAEKGPGQGPGGDG HQESLPPPEFSKDSGFLEELPEDNLSSWATWGTLPPEPNLVPGGPPVSLQTLTFCWESSP EEEEEARESEIEDSDAGSWGAESTQRTEDRGRTLGHYMAR |
| **IL-29/IFNL1** | Q8IU54 | MAAAWTVVLVTLVLGLAVAGPVPTSKPTTTGKGCHIGRFKSLSPQELASFKKARDALEES LKLKNWSCSSPVFPGNWDLRLLQVRERPVALEAELALTLKVLEAAAGPALEDVLDQPLHT LHHILSQLQACIQPQPTAGPRPRGRLHHWLHRLQEAPKKESAGCLEASVTFNLFRLLTRD LKYVADGNLCLRTSTHPEST |
| **IL-30** | Q8NEV9 | MGQTAGDLGWRLSLLLLPLLLVQAGVWGFPRPPGRPQLSLQELRREFTVSLHLARKLLSE VRGQAHRFAESHLPGVNLYLLPLGEQLPDVSLTFQAWRRLSDPERLCFISTTLQPFHALL GGLGTQGRWTNMERMQLWAMRLDLRDLQRHLRFQVLAAGFNLPEEEEEEEEEEEEERKGL LPGALGSALQGPAQVSWPQLLSTYRLLHSLELVLSRAVRELLLLSKAGHSVWPLGFPTLS PQP |
| **IL-31** | Q6EBC2 | MASHSGPSTSVLFLFCCLGGWLASHTLPVRLLRPSDDVQKIVEELQSLSKMLLKDVEEEK GVLVSQNYTLPCLSPDAQPPNNIHSPAIRAYLKTIRQLDNKSVIDEIIEHLDKLIFQDAP ETNISVPTDTHECKRFILTISQQFSECMDLALKSLTSGAQQATT |
| **IL-32** | P24001 | MCFPKVLSDDMKKLKARMVMLLPTSAQGLGAWVSACDTEDTVGHLGPWRDKDPALWCQLC LSSQHQAIERFYDKMQNAESGRGQVMSSLAELEDDFKEGYLETVAAYYEEQHPELTPLLE KERDGLRCRGNRSPVPDVEDPATEEPGESFCDKVMRWFQAMLQRLQTWWHGVLAWVKEKV VALVHAVQALWKQFQSFCCSLSELFMSSFQSYGAPRGDKEELTPQKCSEPQSSK |
| **IL-33** | O95760 | MKPKMKYSTNKISTAKWKNTASKALCFKLGKSQQKAKEVCPMYFMKLRSGLMIKKEACYF RRETTKRPSLKTGRKHKRHLVLAACQQQSTVECFAFGISGVQKYTRALHDSSITGISPIT EYLASLSTYNDQSITFALEDESYEIYVEDLKKDEKKDKVLLSYYESQHPSNESGDGVDGK MLMVTLSPTKDFWLHANNKEHSVELHKCEKPLPDQAFFVLHNMHSNCVSFECKTDPGVFI GVKDNHLALIKVDSSENLCTENILFKLSET |
| **IL-36 α** | Q9UHA7 | MPRGFTWLRYLGIFLGVALGNEPLEMWPLTQNEECTVTGFLRDKLQYRSRLQYMKHYFPI NYKISVPYEGVFRIANVTRLQRAQVSERELRYLWVLVSLSATESVQDVLLEGHPSWKYLQ EVETLLLNVQQGLTDVEVSPKVESVLSLLNAPGPNLKLVRPKALLDNCFRVMELLYCSCC KQSSVLNWQDCEVPSPQSCSPEPSLQYAATQLYPPPPWSPSSPPHSTGSVRPVRAQGEGL LP |
| **IL-36 β** | Q9NZH7 | MNPQREAAPKSYAIRDSRQMVWVLSGNSLIAAPLSRSIKPVTLHLIACRDTEFSDKEKGN MVYLGIKGKDLCLFCAEIQGKPTLQLKLQGSQDNIGKDTCWKLVGIHTCINLDVRESCFM GTLDQWGIGVGRKKWKSSFQHHHLRKKDKDFSSMRTNIGMPGRM |

| | | |
|---|---|---|
| **IL-36 γ** | Q9NZH8 | MRGTPGDADGGGRAVYQSMCKPITGTINDLNQQVWTLQGQNLVAVPRSDSVTPVTVAVIT<br>CKYPEALEQGRGDPIYLGIQNPEMCLYCEKVGEQPTLQLKEQKIMDLYGQPEPVKPFLFY<br>RAKTGRTSTLESVAFPDWFIASSKRDQPIILTSELGKSYNTAFELNIND |
| **IL-37** | **Q9NZH6** | MSFVGENSGVKMGSEDWEKDEPQCCLEDPAGSPLEPGPSLPTMNFVHTSPKVKNLNPKKF<br>SIHDQDHKVLVLDSGNLIAVPDKNYIRPEIFFALASSLSSASAEKGSPILLGVSKGEFCL<br>YCDKDKGQSHPSLQLKKEKLMKLAAQKESARRPFIFYRAQVGSWNMLESAAHPGWFICTS<br>CNCNEPVGVTDKFENRKHIEFSFQPVCKAEMSPSEVSD |
| **IL-38** | **Q8WWZ1** | MCSLPMARYYIIKYADQKALYTRDGQLLVGDPVADNCCAEKICILPNRGLARTKVPIFLG<br>IQGGSRCLACVETEEGPSLQLEDVNIEELYKGGEEATRFTFFQSSSGSAFRLEAAAWPGW<br>FLCGPAEPQQPVQLTKESEPSARTKFYFEQSW |
| **IL-40** | **Q6UX52** | MGLPGLFCLAVLAASSFSKAREEEITPVVSIAYKVLEVFPKGRWVLITCCAPQPPPPITY<br>SLCGTKNIKVAKKVVKTHEPASFNLNVTLKSSPDLLTYFCWASSTSGAHVDSARLQMHWE<br>LWSKPVSELRANFTLQDRGAGPRVEMICQASSGSPPITNSLIGKDGQVHLQQRPCHRQPA<br>NFSFLPSQTSDWFWCQAANNANVQHSALTVVPPGGDQKMEDWQGPLESPILALPLYRSTR<br>RLSEEEFGGFRIGNGEVRGRKAAAM |
